# Understanding Graphene Response to Neutral and Charged Lead Species: Theory and Experiment

**DOI:** 10.3390/ma11102059

**Published:** 2018-10-22

**Authors:** Ivan Shtepliuk, Maria Francesca Santangelo, Mikhail Vagin, Ivan G. Ivanov, Volodymyr Khranovskyy, Tihomir Iakimov, Jens Eriksson, Rositsa Yakimova

**Affiliations:** 1 Department of Physics, Chemistry and Biology, Linköping University, SE-58183 Linköping, Sweden; maria.francesca.santangelo@liu.se (M.F.S.); mikhail.vagin@liu.se (M.V.); ivan.gueorguiev.ivanov@liu.se (I.G.I.); volkh@ifm.liu.se (V.K.); tihomir.iakimov@liu.se (T.I.); jens.eriksson@liu.se (J.E.); rositsa.yakimova@liu.se (R.Y.); 2 Frantsevich Institute for Problems of Materials Science, NASU, 142 Kyiv, Ukraine; 3 Department of Science and Technology, Physics and Electronics, Linköping University, SE-58183 Linköping, Sweden

**Keywords:** lead, electrochemical detection, conductometric detection, sensing mechanism, DFT, epitaxial graphene

## Abstract

Deep understanding of binding of toxic Lead (Pb) species on the surface of two-dimensional materials is a required prerequisite for the development of next-generation sensors that can provide fast and real-time detection of critically low concentrations. Here we report atomistic insights into the Lead behavior on epitaxial graphene (Gr) on silicon carbide substrates by thorough complementary study of voltammetry, electrical characterization, Raman spectroscopy, and Density Functional Theory (DFT). It is verified that the epitaxial graphene exhibits quasi-reversible anode reactions in aqueous solutions, providing a well-defined redox peak for Pb species and good linearity over a concentration range from 1 nM to 1 µM. The conductometric approach offers another way to investigate Lead adsorption, which is based on the formations of stable charge-transfer complexes affecting the *p*-type conductivity of epitaxial graphene. Our results suggest the adsorption ability of the epitaxial graphene towards divalent Lead ions is concentration-dependent and tends to saturate at higher concentrations. To elucidate the mechanisms responsible for Pb adsorption, we performed DFT calculations and estimated the solvent-mediated interaction between Lead species in different oxidative forms and graphene. Our results provide central information regarding the energetics and structure of Pb-graphene interacting complexes that underlay the adsorption mechanisms of neutral and divalent Lead species. Such a holistic understanding favors design and synthesis of new sensitive materials for water quality monitoring.

## 1. Introduction

Lead, belonging to the category of heavy metals, is one of the most dangerous substances that adversely affects intracellular biochemical processes in living creatures [1,2,3,4,5]. The negative impact on human health is originating from its significant toxicity [6] and ability to accumulate in the body [7]. As a result, the normal functions of the relevant organs and tissues are disrupted, causing poisoning and even deaths in some cases (when critical concentrations are exceeded) [8,9]. The mechanisms of Lead toxicity are studied in detail at cellular and subcellular levels [10,11]. Particularly, the Pb^2+^ ions tend to bind to the sulfhydryl, phosphate, and carboxyl groups of the cell membrane, increase its rigidity and reduce the resistance to osmotic shock [12,13,14]. A correlation between Lead concentration in bones and the frequency of development of nephropathy and neurophysiological disorder was also found [15,16]. Nevertheless, the correct determination of the toxicity mechanisms is often complicated by the existence of various ways of penetration of Lead ions into the human body. Lead-containing contaminants can be inhaled; penetrate through the skin; and be ingested through food and drinking water. In view of the foregoing, the need for deep understanding how to detect extra-low Lead concentrations is of relevance.

Conventionally, Lead concentrations are usually determined using one of two the most popular techniques: atomic absorption spectroscopy (AAS) [17] and inductively coupled plasma mass spectrometry (ICPMS) [18]. On one hand, these methods provide accurate measurements of Pb in serum and blood. On the other hand, they require time-consuming preparation and analysis, expensive/extensive equipment, and highly qualified personnel. Moreover, significant time delays due to the delivery of samples to centralized laboratories make these methods less desirable and even unsuitable for real-time analysis. Such shortcomings stimulated the development of portable and easy-to-use methods of detection. Since the 1970s, when the first anodic stripping voltammetry measurements of Lead concentrations in natural and ground waters were reported [19,20,21], the development of electrochemical methods for the trace Lead analysis in aqueous solutions has significantly improved [22,23,24]. Less attention has been paid to the development of conductometric (also known as resistive) sensors for Lead analysis [25,26,27]. Unlike the so-called “bulky” techniques, the electrochemical (anodic stripping voltammetry, cyclic voltammetry and chronoamperometry) and conductometric (resistance-time measurements) techniques for Lead determination use simple equipment (in most cases three-electrode electrochemical cell and resistor/transistor, respectively) and promote further miniaturization of detection devices. Such methods primarily allow for precise identification of the type of the heavy metal and to control drinking water quality in accordance with the WHO safe limit for Pb of 10 ppb [28]. The efficiency of these methods is unambiguously dependent on the choice of the optimally sensitive material, which must possess a large active surface area, a necessary potential window for Pb-involved redox reactions for effective electrochemical detection and high conductivity for high-performance conductometric analysis, respectively. In this context, deep understanding of the adsorption/interaction mechanism of Lead at/with electrode surface is a required prerequisite for designing high-performance Lead detection devices.

Despite the huge progress that has been made towards high-precision electrochemical methods for discriminative Lead analysis, the development of new approaches and advances in novel materials are still highly demanded acting as driving forces for the proliferation of next-generation sensing applications from laboratory to smart at-home heavy metal test kits. Thus, it is not surprising that atomically thin two-dimensional materials such as graphene are proving to be a popular platform for creating ultra-sensitive sensors, which are able to feel even individual adsorbates [29]. Since the discovery of graphene in 2004 [30], many research groups attempted to implement graphene-based materials to electrochemical sensors for real-time detection and quantification of Lead [31,32,33,34,35,36,37,38,39,40,41,42,43,44]. Existing literature data suggest that modification of the working electrodes by (reduced) graphene oxide, graphene quantum dots and graphene-containing nanocomposites is the most popular way to improve the sensitivity of traditional working electrodes towards Lead detection. It was argued that the presence of graphene in the nanocomposite can facilitate the electron transfer rate during Pb-involved redox reactions and causes higher anodic peak current density, which is due to the high conductivity of graphene [32]. Furthermore, it was reported that the existence of functional groups with large negative charges (mainly –OH and –COOH) on graphene surface is very favorable for effective adsorption of Pb^2+^ cations [45]. Nevertheless, in most cases, highly reactive graphene derivatives are toxic materials [46] and their using implies not only enhancement of the sensitivity towards Pb detection, but also may have negative impact on environmental health and safety. In this regard, using the working electrode materials, which exploit only the key properties of unmodified and non-toxic graphene would be more desirable.

Among different graphene family materials, epitaxial graphene, having versatile properties beyond graphite and exfoliated graphene [47], can become a sensing material of choice in the future. The appeal of epitaxial graphene for detection of heavy metals lies in its large surface area (providing large number of electroactive sites) [48], high chemical stability (no transfer is needed for sensors fabrication, since the graphene is naturally supported by a SiC substrate) [49], wide potential window [50], biocompatibility [51], high carrier mobility [52] and tunable electrochemical activity [53]. Such properties are excellent prerequisites to exploit epitaxial graphene as both working electrode and conducting material towards reliable and real-time detection of Lead in aqueous solutions. In this context, fundamental knowledge about adsorption/interaction mechanisms is highly beneficial. In our previous work [50] we reported on the electrodeposition of Lead on epitaxial graphene and showed that the initial kinetics of Pb is governed by diffusion-controlled instantaneous nucleation mechanism (with quite small diffusion coefficient). In principle, such a behavior provides favorable conditions for strong adsorption of Pb. Nevertheless, the mechanisms underlying the adsorption of Lead species in different oxidation forms on epitaxial graphene are still not fully understood. The objective of the present work is twofold: (i) to shed light on the redox behavior of Pb at the epitaxial graphene electrode and (ii) to understand the fundamental difference in the adsorption mechanisms of neutral and oxidized Pb species. In particular, we are aiming to investigate the electrochemical activity of Gr/SiC towards Lead by two different approaches: voltammetry and resistance-time measurements. Intuitively it seems that interaction strength between graphene and reduced metallic species (elemental Pb^0^) can be determined by using voltammetric methods through monitoring the Pb-involved redox process, while direct adsorption of divalent Pb ions can be investigated by measuring the resistance changes of epitaxial graphene under exposure to Pb-containing liquid. In fact, we showed recently that the binding energy of Pb^2+^ on graphene immersed in water is higher than that of neutral Pb atom [54]. Therefore, we are interested in determining whether changes in the charge state of Pb species could influence the adsorption mechanism. The special focus will be placed on the theoretical investigation of the nature of t interaction between Lead species and graphene by using density functional theory calculations. We anticipate that the combined theoretical and experimental study of the metal-graphene interacting complex will provide holistic understanding of Lead behavior on graphene surface at an atomistic level and will be very useful for designing graphene-based materials with high adsorption capacity and sensitivity to Pb species dissolved in aqueous solutions.

## 2. Materials and Methods

### 2.1. Synthesis and Characterization of the Epitaxial Graphene

Epitaxial graphene on on-axis SiC (Gr/SiC) was synthesized by sublimating Si atoms from SiC substrates at high temperature [55]. Thermal decomposition of the semi-insulting Si-face (0001) 4H-SiC substrate (7 × 7 mm^2^) occurred in argon atmosphere using an inductively heated graphite container with well controlled temperature profile. Optical reflectance mapping [56] indicates that monolayer (1 ML) graphene (more than 70% of the total area) covers most of the template surface, while the bilayer graphene inclusions are also observed (less than 30%). The quality of as-grown epitaxial graphene was investigated by Raman spectroscopy. No *D*-line which characteristic of point defects is was observed on the pristine graphene sample. The Raman spectra were collected by means of a micro-Raman setup based on a monochromator (Jobin-Yvon, model HR460, Longjumeau, France) equipped with a CCD (couple-charged device) camera. The objective lens has a magnification of 100× and numerical aperture NA = 0.95 resulting in a ~0.85 μm diameter of the laser spot focused on the sample surface. A 532 nm diode-pumped solid-state laser with 17 mW power was used as an excitation source. The spectral resolution of the system is ~5.5 cm^−1^.

### 2.2 Electrochemical Measurements

Computer-controlled potentiostat (Autolab, EcoChemie, Metrohm, Utrecht, The Netherlands) was used in all room-temperature electrochemical measurements. The laboratory-made electrochemical cell of o-ring type was coupled with working electrode (Gr/SiC anode), reference electrode (Ag/AgCl) and counter electrode (platinum wire). More details on the design of the electrochemical cell can be found in our previous work [53]. At the first stage, to better understand the redox behavior of Lead we performed cyclic voltammetry measurements within a negative potential region from −0.9 V to 0 V in buffer solution (0.1 mol·L^−1^ HClO_4_ in Milli-Q-water) with 0.1 mM Pb^2+^ (purity of Pb(NO_3_)_2_ is higher than 99%). The scan rate was 20 mV/s. The choice of aqueous perchlorate solution as buffer solution is justified by the fact that the perchlorate ions (ClO^4−^) exhibit non-complexing character with respect to metal cations in aqueous solution [57,58,59]. It means that the behavior of Lead species is only related to Pb-involved oxidation-reduction reactions, but not to the reactions involving more complicated chemical complexes. At the next stage, aqueous solutions containing different concentrations of Pb^2+^ were added into the electrochemical cell and the resulting solution was stirred at the potential of −1.2 V for 2 min. Then a stripping process was performed by square wave anodic stripping voltammetry (SWASV) over the potential range from −1.2 V to −0.4 V at the following parameters: frequency, 15 Hz; amplitude, 25 mV; increment potential, 5 mV. To prevent losses caused by the Lead adsorption on the container walls, we carefully washed the electrochemical cell with weakly concentrated nitric acid before each electrochemical test and adjusted the pH of the aqueous solution to the value of 4.5.

### 2.3. Conductometric Measurements

To investigate conductometric regime we use epitaxial graphene as a lateral chemiresistor, which can change its resistance under exposure to the toxic Lead ions (within concentration range from 125 nM to 500 µM) in aqueous solutions. Titanium/gold contact metallization (2/200 nm thick) is added on the graphene surface using standard sputtering technique. Next, the real-time response of epitaxial graphene resistor (with four contacts) to liquid-phase water/Pb mixture was examined. For this aim, we record the electrical resistance of the graphene film during exposure/recovery cycles. In other words, the current (at fixed bias voltage, 3 V) across the Ti/Au-graphene-Au/Ti interface was measured as a function of time before and after injection of Pb-containing solution in a 3D printed microfluidic device. Electrical resistance of the epitaxial graphene was measured using a 2601A Keithley Source Meter.

### 2.4. Density Functional Theory (DFT) Calculations

To elucidate the nature of the interaction between graphene and Pb species, we performed DFT calculations. All calculations were conducted in Gaussian 09 Rev. D.01 program package [60]. Since neutral Lead species are expected to interact with graphene through the attractive van der Waals forces, the equilibrium Pb adsorption configurations were investigated at PBE1PBE-D3 level of restricted DFT, which includes the empirical dispersion correction [61] with a 6-31G(d) basis set for carbon and hydrogen atoms as well as a basis set developed by the Stuttgart–Dresden–Bonn group (SDD) for the Lead species [62]. As has been demonstrated in our previous works [63,64,65], such a combination of the method and basis sets gives a possibility to predict van der Waals interaction between elemental heavy metals and graphene. On the other hand, electrostatic interaction between divalent charged Lead species and graphene can be estimated by using B3LYP (Becke, three-parameter, Lee-Yang-Parr) exchange-correlation functional [66]. In principle, the metal binding to graphene should be modelled by using the periodic boundary conditions (PBC) calculations replicating the characteristics of an infinite honeycomb structure of graphene. However, the models exploiting polycyclic aromatic hydrocarbons (PAHs) may also adequately describe the local adsorption phenomena. To prevent the effect of highly reactive edges on the adsorption of metal species, a structure with a large number of carbon atoms, namely C_96_H_24_ (circumcircumcoronene [67]) was chosen. To investigate the role of aqueous solution all calculations were conducted in the presence of water medium by using polarizable continuum model (PCM) [68]. Geometry optimization calculations were performed with SCF (self-consistent field) convergence criterion of 10^−8^. Mulliken population analysis [69] and Hirshfeld scheme [70] were applied to study the charge distribution within interacting complexes. We estimated the adsorption (*E*_ads_), interaction (*E*_int_) and deformation (*E*_def_) energies for the most favorable geometrical configurations of Lead species, which can be represented by the following expressions:

(1) $$\{\begin{array}{c} E_{\mathrm{ads}}=\left(E_{\mathrm{tot}}^{\mathrm{iso}-{\mathrm{Pb}}^{2+/0}}+E_{\mathrm{tot}}^{\mathrm{iso}-G}\right)-E_{\mathrm{tot}}^{\mathrm{rel}-{\mathrm{Pb}}^{2+/0}@G}\\ E_{\mathrm{int}}=\left(E_{\mathrm{tot}}^{\mathrm{rel}-{\mathrm{Pb}}^{2+/0}}+E_{\mathrm{tot}}^{\mathrm{rel}-G}\right)-E_{\mathrm{tot}}^{\mathrm{rel}-{\mathrm{Pb}}^{2+/0}@G}\\ E_{\mathrm{def}}=E_{\mathrm{int}}-E_{\mathrm{ads}} \end{array}$$

where $E_{\mathrm{tot}}^{\mathrm{rel}-{\mathrm{Pb}}^{2+/0}@G}$ is the total energy of the graphene after complexation with Pb^2+/0^, $E_{\mathrm{tot}}^{\mathrm{iso}-{\mathrm{Pb}}^{2+/0}}$ and $E_{\mathrm{tot}}^{\mathrm{iso}-G}$ are the total energies of non-relaxed isolated Lead ion and graphene, respectively. While $E_{\mathrm{tot}}^{\mathrm{rel}-{\mathrm{Pb}}^{2+/0}}$ and $E_{\mathrm{tot}}^{\mathrm{rel}-G}$ are the total energies of the Lead ion and graphene in the relaxed geometry. To investigate the stability of the chemisorbed Pb^2+^ ions on graphene we also performed ab-initio molecular dynamic calculations at room temperature. Potential energy trajectory was obtained by atom density matrix propagation (ADMP) method [71] implemented in Gaussian 09 Rev. D.01 program package with a time step (Δ*t*) of 0.1 fs.

## 3. Results

### 3.1. Properties of the Epitaxial Graphene Electrode

**Figure 1:**
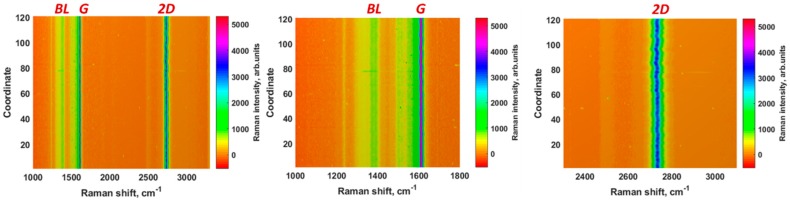
Two-dimensional contour plots of Raman spectra of as-grown epitaxial graphene conveying both the intensity and spectral uniformity of the predominant vibrational *G* and 2*D* modes. The whole Raman fingerprint of the epitaxial graphene and the regions zooming near the *G* and 2*D* peaks are depicted in the three panels from left to right, respectively.

Before focusing on the principles underlying the redox behavior of Lead at epitaxial graphene, we investigated vibrational properties of as-grown samples by Raman spectroscopy with the aim to address the question on stability of the electrode material. Indeed, from the practical point of view it is very important to use the working electrode having understandable and reproducible properties. In this context, Raman measurements can be used to give quick feedback on the quality of graphene samples, including defect density, number of graphene sublayers, homogeneity and so on. To test whether the epitaxial graphene is uniform enough to be used as working electrode, we collected a set of Raman spectra on a square map (3 µm × 3 µm, 0.3 µm pitch). Figure 1 (left panel) exhibits the two-dimensional color-coded plots of the set of 121 Raman spectra (see also the corresponding zoomed spectral regions in the middle and right panels). It is clearly seen that the intensity over 121 spectra is uniform and only two main peaks (so-called *G* and 2*D* modes) and the weak structured band related to the buffer layer (BL) are distinguishable. We do not detect any Raman signal from the defect-related *D*-mode, demonstrating the high crystalline quality of the epitaxial graphene. 

Simple statistical analysis on the set of 121 Raman spectra was used to estimate the uniformity of the Raman scattering intensity. We calculated the relative standard deviation (RSD) of intensities of the characteristic *G* and 2*D* Raman modes. RSD values for these peaks are found to be 3.2% and 3.7%, respectively, indicating that the as-grown epitaxial graphene possesses excellent Raman scattering uniformity. Taking the aforementioned into account, we anticipate that sensing performance of epitaxial graphene working electrode will be determined by the direct interaction between analyst (Lead species in our case) and defect-free *sp*^2^ conjugated graphene domains. More details on the vibrational properties of the as-grown epitaxial graphene can be found in our recent work [50].

### 3.2. Electrochemical Activity of Epitaxial Graphene towards Lead

**Figure 2:**
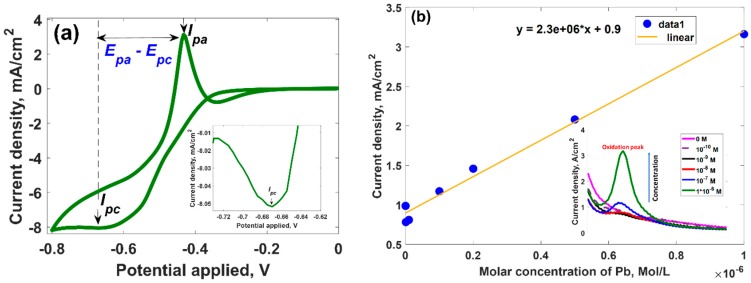
(**a**) Cyclic voltammogram response of 0.1 mM Pb^2+^ in 0.1 M HClO_4_ at pH 4.5 with scan rate of 20 mV∙s^−1^ at epitaxial graphene. Inset shows zoomed region of the cyclic voltammogram, which represents the cathodic process (**b**) Calibration curve showing the dependence of the current density (maximum of the oxidation peak) at the molar concentration of Lead in solution. Inset demonstrates SWASV responses of epitaxial graphene at different concentrations of Pb^2+^ ions.

We investigated the electrochemical response of the epitaxial graphene to Pb-involved reduction-oxidation reactions by using cyclic voltammetry (CV) technique. Such characterization is very helpful tool to understand deeply the nature of redox behavior of Pb in the presence of graphene. A typical cyclic voltammogram recorded for 0.1 mM Pb^2+^ in 0.1 M HClO_4_ with scan rate of 20 mV∙s^−1^ at pH 4.5 is demonstrated in Figure 2. 

It is obvious that two distinguishable peaks related to the consecutive redox couple of Pb^2+^/Pb^0^ are present: in the forward scan a cathodic peak (reduction process of Pb^2+^ to Pb^0^), *I*_pc_ at −0.669 V, and in the reverse scan an intense anodic peak (oxidation process of Pb^0^ to Pb^2+^), *I*_pa_ at −0.433 V. This implies that Pb^2+^ undergoes two-electron transfer redox reaction. The possible mechanism of the redox reaction can be described by the following equilibrium reactions:

(2) $$\{\begin{array}{c} {\mathrm{Pb}}^{2+}+2e\to {\mathrm{Pb}}^{0}\;\left(\mathrm{reduction}:\;\mathrm{cathodic}\;\mathrm{process}\right)\\ {\mathrm{Pb}}^{0}-2e\to {\mathrm{Pb}}^{2+}\;\left(\mathrm{oxidation}:\;\mathrm{anodic}\;\mathrm{process}\right) \end{array}$$

To shed more light on the electroactive behavior of epitaxial graphene to Pb species, we estimated the potential difference (Δ*E_p_*) between cathodic and anodic peaks and peak current ratio. It was revealed that the peak-to-peak separation Δ*E_p_* and the ratio of redox peak current *I*_pa_/*I*_pc_ are about 236 mV and 3.67, respectively, indicating that Pb-involved redox process at the surface of epitaxial graphene is an electrochemically quasi-reversible process [68]. In principle, having these parameters determined it is possible to predict the diffusion coefficients of the electroactive species and electron transfer rate constants for both oxidation and reduction process. The fundamental relationship between anodic/cathodic current and corresponding diffusion coefficient in the case of quasi-reversible system at 298 K can be described by using Randles–Ševcik equations [72,73]:

(3) $$I_{p,c}^{quasi}=-\left(2.65\times 10^{5}\right)n^{3/2}ACD_{c}^{1/2}\nu^{1/2}$$

(4) $$I_{p,a}^{quasi}=+\left(2.65\times 10^{5}\right)n^{3/2}ACD_{a}^{1/2}\nu^{1/2}$$

where $I_{p,c}^{quasi}$ and $I_{p,a}^{quasi}$ is the cathodic and anodic peak current densities in A∙cm^−2^, *D_a_* and *D_c_* are the diffusion coefficients of oxidative and reduced species in cm^2^∙s^−1^, *C* is the bulk concentration of oxidative species in solution, *v* is scan rate in V∙s^−1^, *n* is the number of electrons transferred and *A* is the area of the working electrode. In the case when (Δ*E_p_*) > 200 mV, the electron transfer rate constant may be determined by using Klingler and Kochi relationship [74]:

(5) $$k^{0}=2.18\left[D\alpha n\nu F/{\left(RT\right)}^{1/2}\right]\exp\left[-\left(\alpha^{2}nF/RT\right)\Delta E_{P}\right]$$

where *F* is Faraday constant, *R* is gas constant, *T* is a temperature and *α* is transfer coefficient (typically a value of 0.5 is assumed). The calculated parameters are summarized in Table 1. It is clearly seen that the rate of diffusion of reduced species is slower than that for the oxidized species. Using Equation (5), the value of rate of the electron transfer from the electrode to Pb^2+^ was estimated to be higher than the rate of electron transfer from the Pb^0^ species to the electrode surface. Due to this reason, the corresponding oxidative peak current increased more rapidly with increasing the concentration of the Lead species in solution in comparison to the reduction peak current. Therefore, the oxidation peak was chosen as the analytic signal for more detailed quantification of the Lead species. To investigate the stability of the graphene electrode towards Pb redox reactions, we repeated cyclic voltammetry measurements over 10 cycles (not shown here) and revealed that CV curves are almost identical with clear oxidations peaks and without any potential shift. The % RSD for 10 repetitions analysis in 0.1 M HClO_4_ containing 0.1 mM Pb^2+^ was 3.9%. Altogether this means that the epitaxial graphene electrode shows good stability and reproducibility towards Pb electrodeposition.

To monitor the dependence of the analytic signal on the concentration of the Lead ions in solution, we performed more effective SWASV analysis (since the CV analysis is typically limited to μM concentrations because of large sensitivity of this method to charging current) for Pb in the concentration range between 10^−10^ M to 10^−6^ M. Figure 2b (see inset) shows the SWASV response of epitaxial graphene in the presence of Lead-containing solution. It can be seen the appearance of single, well-defined peak and the stripping peak current was proportional to the concentration of Pb^2+^. The linear regression curve in Figure 2b indicates that the sensitivity of the epitaxial graphene to Pb is 2.3·10^3^ A·cm^−2^·mole^−1^·L, with good linearity (*R* = 0.9945). The correlation equation is *I* (mA/cm^2^) =2.3 × 10^6^·[Pb(mole/L)] + 0.9. The RSD of Pb determination with six repetitions was 3.5%.

### 3.3. Mechanism of the Electrochemical Response of Epitaxial Graphene towards Lead

To gain deep insight into the adsorption mechanism, it is very important to clarify the fundamental principle of generation of the stripping current observed in our experiment. Generally, SWASV comprises two stages: (i) preconcentration step and (ii) stripping step. During the first preconcentration step, Lead divalent ions (Pb^2+^) are initially adsorbed on the inert electrode surface and then they are reduced to neutral Lead species (elemental Pb^0^) at a constant potential. At the second stage, Pb^0^ species are re-oxidized to Pb^2+^ and desorbed back to the solution in the oxidized state. During the stripping of Lead species accumulated onto the epitaxial graphene surface, Pb^0^ adatoms lose 2 electrons, thereby contributing to the generation of the stripping current. It is important to note that the resulting stripping current is proportional to the concentration of the adsorbed species. This means that the preconcentration process predominantly defines the sensitivity of the epitaxial graphene towards Pb. Indeed, the more Pb^2+^ ions will be plated as neutral Pb species on the electrode, the more will be re-oxidized and thus the stronger the striping peak current is expected. Therefore, a knowledge about the interaction between elemental Lead species and graphene in the presence of water is a key to understanding the sensing mechanism at the atomistic level. For this aim we performed comprehensive DFT calculations focusing on the nature of this interaction. Since the van der Waals (vdW) forces are expected to play a fundamental role in the adsorption of elemental metals on graphene [75], we compared two different schemes—PCM/B3LYP/6-31G(d)/SDD and vdW-corrected PCM/PBE1PBE-D3/6-31G(d)/SDD—in order to estimate the energy contribution of dispersion forces to total interaction energy. DFT-calculated parameters describing the adsorption of Pb^0^ on graphene nanofragment are summarized in Table 2.

**Figure 3:**
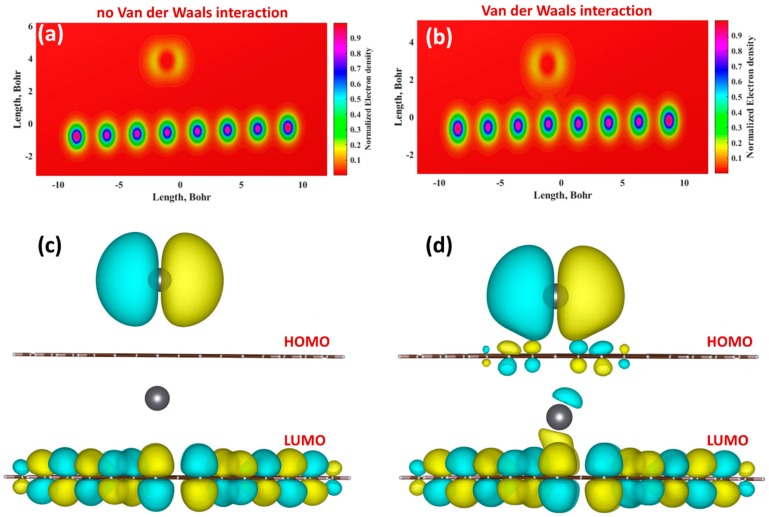
Color-filled maps of electron density of Pb@graphene system without (**a**) and with (**b**) vdW correction. Spatial distribution of wave functions corresponding to the HOMO and LUMO orbitals of Pb@graphene system without (**c**) and with (**d**) vdW correction. The yellow and cyan colors indicate positive and negative phases in the wave function, respectively. The orbitals are drawn at an iso-surface value of 0.02.

To explore the effect of van der Waals forces on the geometry structure of adsorption configuration, we first examined the stability of the adsorption sites and equilibrium binding distances. In both cases, neutral Pb species tend to occupy the bridge site (above the center of C-C bond). According to B3LYP results, binding height of Pb adatom is 4.36 Å (see Table 2). By considering the vdW correction, the equilibrium binding distance is decreased to 3.18 Å by 1.18 Å. The large reduction in the equilibrium separation between Pb and graphene causes a strong hybridization between the Pb-related orbitals and graphene *p*_z_ orbitals. Additional evidence of such hybridization was obtained from the analysis of the color-filled maps of electron density and orbital wave functions (see Figure 3) without and with vdW correction, respectively. Figure 3a shows that without vdW correction, the charge transfer from graphene to Pb is negligibly small and therefore the wave functions corresponding to HOMO (highest occupied molecular orbital) and LUMO (lowest occupied molecular orbital) orbitals are completely localized on Pb adatom and delocalized over the graphene plane, respectively (as demonstrated in Figure 3c). However, when we consider the vdW correction (Figure 3b), we notice that the electron density increases between *sp*^2^ plane and metal adatom and electron clouds are deformed due to Pb-C orbital hybridization. 

Furthermore, an important role of London dispersion forces in interaction between Pb and graphene is also evidenced by the spatial redistribution of electron wavefunctions describing frontier molecular orbitals (see Figure 3d). In particular, it is clearly seen that both HOMO and LUMO are shared between Pb and graphene. We also compared the interaction energies between Pb and graphene in the presence of solvent with and without consideration of the van der Waals forces. The interaction energy of Pb without vdW correction has a very small negative value, indicating the dominant role of repulsive forces. This energy is significantly increased from ~−5 meV to 324 eV after considering the vdW correction (Table 2). It is obvious that the additional van der Waals interaction strengthens the total Pb-graphene interaction by 329 meV. As mentioned before, we also calculated the adsorption energy to define the energy paid to deform the graphene plane during the interaction process. Our estimations suggest that the difference between interaction energy and adsorption energy is very small and graphene undergoes no observable deformation upon adsorption event. In this regard, the vdW-enhanced adsorption of Pb onto graphene immersed in water can be classified rather as physisorption than chemisorption. It is noteworthy that in both cases Pb species immersed in aqueous medium act as electron acceptors and thus the direction of the charge transfer is from graphene to metal. According to Mulliken and Hirshfeld analyses, when we consider the vdW correction the charge transfer is increased (see Table 2). As a result, we also observe a significant increase in the dipole moment along *z*-direction (perpendicular to the graphene plane). Therefore, consideration of the vdW correction is a key for gaining insight into the interaction between elemental Pb and graphene and, consequently, for correct explanation of the adsorption mechanism.

**Figure 4:**
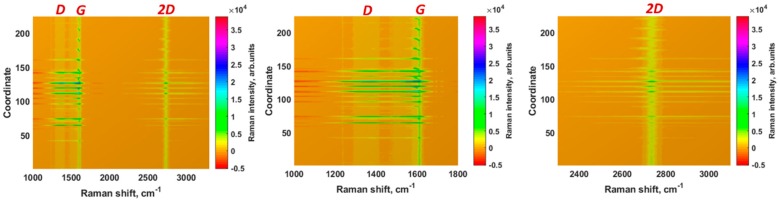
Two-dimensional contour plots of Raman spectra of epitaxial graphene after Pb electrodeposition conveying deteriorated intensity and spectral uniformity of the predominant vibrational modes *G* and 2*D*. Similar to Figure 1, the three panels from left to right show the whole Raman fingerprint after Pb electrodeposition and the regions zooming near the *G* and 2*D* peaks, respectively.

To gain deeper insight into the interaction between elemental Pb and graphene, we also performed Raman mapping. Bearing in mind that as-grown epitaxial graphene exhibits no defect-related *D* peak, we further investigate the Raman spectra of epitaxial graphene after electrodeposition of the Pb to ensure that adsorption capacity is high enough to provide the appropriate stripping response. Detailed analysis of the Pb electrodeposition on epitaxial graphene was performed in our previous work [50]. Therefore, in this work we intended only to visualize the degree of occupation of graphene surface with Lead species. As can be seen from Figure 4, the Pb deposition causes an inhomogeneous distribution of the Raman scattering (intensity and spectral positions of the features), as well as appearance of the *D*-line. Since the *D*-mode was not present before electrodeposition process, it is reasonable to assume that its appearance is due to the formation of Pb-C bonds.

Furthermore, the observed non-uniform *D* peak intensity distribution suggests that the perturbation related to adsorption of Pb species has local character, most likely restricted to the vicinity of the adsorbed species. Since large Pb clusters are weakly bonded to graphene [50], it is more likely that only randomly distributed individual Pb adatoms and small Pb nano-clusters could affect the vibrational properties of graphene leading to the appearance of the *D* peak. To summarize this section, one can conclude that epitaxial graphene provides enough available electroactive sites for oxidation-reduction reactions during stripping analysis.

### 3.4. Conductometric Response of Epitaxial Graphene towards Pb

**Figure 5:**
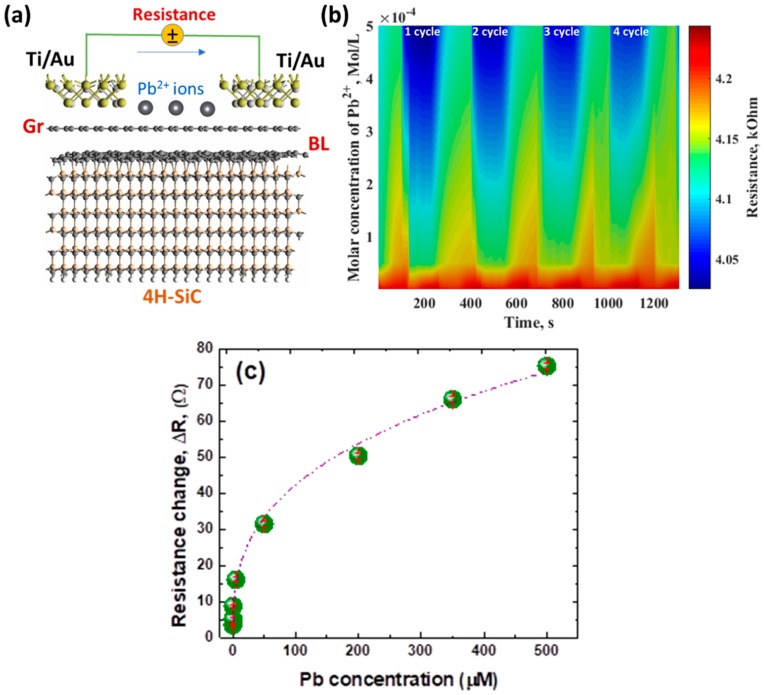
(**a**) Schematic representation of measurement principle of the real-time monitoring of the resistance of the epitaxial graphene under Pb^2+^ exposure. (**b**) Two-dimensional contour plot of the electrical response of epitaxial graphene recorded during adsorption-desorption processes at the varying concentrations of Pb^2+^ in water electrolyte. (**c**) Dependence of resistance change value, Δ*R*, on the concentration of Lead.

As was mentioned before we used epitaxial graphene as a lateral resistor for measurement of the conductometric response. Schematic representation of the device is depicted in Figure 5a. From this Figure it is clearly seen that the core of such simple device is epitaxial graphene on Si-face 4H-SiC (its atomistic structure contains undisturbed SiC layers and carbon-rich interfacial layer, followed by one graphene layer). At the next stage, titanium/gold ohmic contacts were deposited onto pristine epitaxial graphene. Since the binding height of neutral metals adatoms (both Ti and Au) on graphene is expected to be lower than 2 nm [76], we assumed some distance between metallization and the topmost graphene layer, as illustrated in Figure 5a. By applying a constant voltage to the contacts (3 V), we measure the current across the structure Ti/Au/Gr/Au/Ti. In such lateral geometry, any adsorption of Pb^2+^ ions will influence the band structure of graphene and, consequently, the electron transport through graphene. It means that real-time monitoring of the resistance of the epitaxial graphene as a function of time under exposure to injected liquid containing Pb^2+^ ions will allow estimation of how the epitaxial graphene interacts with divalent Lead ions dissolved in water. 

Figure 5b shows the response of the epitaxial graphene to Pb^2+^ at different molar concentrations within the range from 0.125 μM to 500 μM. The graphene resistor exhibits a good response to Pb^2+^ and reproducibility even after several repeated cycles. The initial resistance of the epitaxial graphene changed with the Pb^2+^ solution injected into the flow chamber. It can be understood that the presence of Pb^2+^ ions dissolved in aqueous solution enhances the conductivity of graphene, as evidenced by the resistance drop during each cycle. The relative variation of the resistance was calculated by equation Δ*R* = (*R*_Pb_ − *R*_EG_)/*R*_EG_, where *R*_Pb_ and *R*_EG_ represent the resistances of the epitaxial graphene after injection of Pb-containing liquid and the initial resistance of the epitaxial graphene, respectively. We noticed that the increase in Lead concentration causes an increase of the differential resistance value, suggesting that the adsorption of Pb^2+^ metal ions on the epitaxial graphene follows the Langmuir isotherm (Figure 5c). This dependence contains two different regions: (i) linear region, in which the differential resistance is linearly increased with increasing the Pb concentration (regime of small Lead concentrations); (ii) quasi-linear region that tends to saturate, in which the differential resistance is only weakly dependent on Pb concentration (regime of large Pb concentration). It means that the adsorption ability of epitaxial graphene to Pb^2+^ is concentration-dependent. According to our estimations the slope of these regions decreases with the increase of Pb^2+^ concentration in the electrolyte solution from 13.90 Ω/µM to 0.10 Ω/µM. This signifies that the larger number of reactive sites is available for adsorption and subsequent sensing of nanomolar concentrations Pb^2+^ rather than the micromolar concentrations. Using single-exponential function model, we analyzed the resistance-time curves at the lowest and largest concentrations of Pb^2+^. Furthermore, it was found that the response/recovery time to the Pb^2+^ concentration of 0.125 μM and 500 μM was approximately 35 s/142 s and 11 s/65 s, respectively. Since the recovery time at the lowest Pb concentrations is longer than that at the largest Pb concentrations, it is reasonable to assume that one needs more time for Lead ions to desorb from the surface of epitaxial graphene. The recovery time corelates with the adsorption energy through an exponential law [77], thereby confirming that the adsorption energy of divalent Lead ions is expected to be the largest at the nanomolar concentrations. 

The reproducibility of the resistance response for epitaxial graphene was estimated by analyzing a full set of Pb^2+^ concentrations ranging from 0.125 μM to 500 μM and the standard deviation of the resistance change values for each concentration was evaluated. The RSD values obtained range from 9% to 1% for the lowest and the highest concentrations, respectively. To investigate the repeatability of the graphene resistors response during the adsorption-desorption process, six cycles of responses to Pb-containing water solution for each analyzed concentration were executed. We revealed that the response levels of the epitaxial graphene are maintained even after repeated cycles, indicating good repeatability. When compared with SWASV technique that exploited epitaxial graphene as a working electrode material, the accuracy of measurements of the conductometric responses is lower.

### 3.5. Mechanism of Conductometric Response of Epitaxial Graphene towards Lead

**Figure 6:**
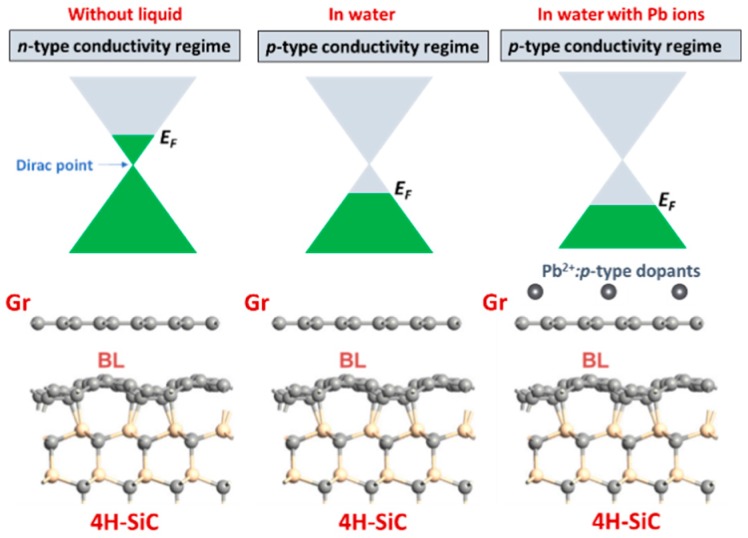
Proposed qualitative model of the response of the epitaxial graphene to the Pb^2+^ ions dissolved in water electrolyte.

Based on our experimental results we can assume that the changes in the resistance of the epitaxial graphene under Pb^2+^ exposure are related to electron transfer dynamics. We propose a model, which explains the conductivity increase (Figure 6a). According to this model, as-grown epitaxial graphene, possessing *n*-type conductivity under ambient conditions, transforms to *p*-type material after adsorption of water molecules on its surface. For this reason, resistance-time measurements towards Lead detection in the presence of water are expected to perform in hole conductivity regime. This implies that a presence of divalent Pb ions in water electrolyte will affect the *p*-type conductivity of epitaxial graphene. Since the positively charged Lead ions behave as typical acceptors one can anticipate a lowering of the Fermi level position with subsequent changes in carrier transport when more and more Pb species adsorb onto graphene. In other words, the graphene donates electrons to metal species, thereby causing the increase in the concentration of holes, which in turn increases the hole conductivity of the epitaxial graphene. This is consistent with our experimental observation of the resistance decrease after injection of electrolyte containing Lead ions.

**Figure 7:**
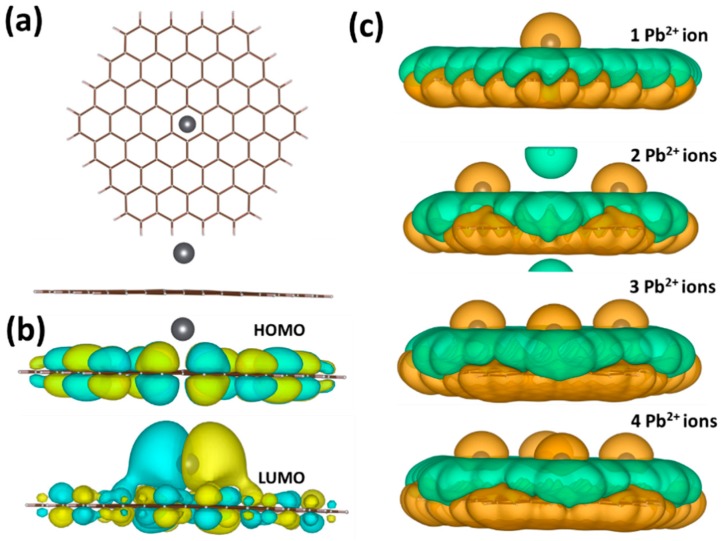
(**a**) Optimized structure of graphene interacted with single Pb^2+^ ion in the presence of water electrolyte. (**b**) Spatial distribution of wave functions corresponding to the HOMO and LUMO orbitals of Pb^2+^@graphene system in solvent. The yellow and cyan colors indicate positive and negative phases in the wave function, respectively. The orbitals are drawn at an iso-surface value of 0.02. (**c**) Charge density difference of the different interacting complexes. The green color represents the source of electrons, and the orange color represents where the electrons are going. The contour iso-value is 0.001.

To better understand the mechanism of Pb^2+^ adsorption at the atomistic level, we have performed DFT calculations and predicted the properties of Pb^2+^@graphene interacting complexes. To quantitively describe the binding character, we investigated four adsorption configurations with different number of divalent Lead species interacting with graphene (from 1 ion to 4 ions) in the presence of solvent (water in our case). It was found that the hollow site (the center of the hexagonal ring) is the most favorable adsorption site of Pb^2+^ ion on graphene (Figure 7a).

Independently of the kind of adsorption configuration, divalent ions always act as typical acceptors, which extract electrons from graphene resulting in the increase in hole concentration. As a result, LUMO is strongly hybridized and shared between graphene and Pb^2+^ ion (Figure 7b), implying the formation of Pb-related unoccupied levels near the Fermi level. While HOMO is completely delocalized over the graphene plane. Both interaction energy and charge transfer per ion depend on the amount of Pb species. The variation of the total charge transferred from graphene to Pb^2+^ ion is less pronounced with values ranging between 1.37*e*^−^ and 1.47*e*^−^ per ion. For the interaction energy, a much stronger dependence was found. In particular, as can be seen from Table 3, with increasing the number of Lead ions the interaction energy per ion decreased from −1.6244 eV (for 1 ion) to −0.3221 eV for (for 4 ions). Similar trend was observed for adsorption energy. Such a decrease in the interaction/adsorption energy implies that adsorption capacity at small and high concentrations of Lead will be different. Higher adsorption capacity is expected at extremely small concentrations of Pb^2+^ in aqueous solution. This theoretical finding is in good agreement with the experimental observation (discussed above) and is very interesting from a practical point of view. As was mentioned before the difference between interaction energy and adsorption energy can be regarded as a degree of deformation of the *sp*^2^-plane adsorption event. With this knowledge in mind, we estimated the deformation energy for all considered adsorption geometries. This parameter was found to increase with increasing number of adsorbates, indicating that more energy penalty needs to be paid to accommodate larger number of adsorbates at the graphene surface. 

To visualize the charge-transfer mechanism, we calculated the charge density difference by using the following equation:

(6) $$\Delta \rho =\rho_{Pb^{2+}@Gr}-\rho_{Gr}-\rho_{Pb^{2+}}$$

where $\rho_{Pb^{2+}@Gr}$, $\rho_{Gr}$, and $\rho_{Pb^{2+}}$ are the total charge densities of the interacting structure, the isolated graphene, and the isolated Pb^2+^ ion, respectively. As shown in Figure 7c, the charge transfer occurs from the graphene to Pb^2+^ ions near the adsorption site. Therefore, hole doping effect is caused by the formation of a hybrid charge-transfer complex. It should also be noted that the charge-transfer value obtained using the vdW-corrected functional is very close to that calculated without vdW correction (Table 3). This suggests that the effect of van der Waals interaction on redistribution of charges in the interacting system in the presence of water is negligibly small. On the other hand, vdW-corrected DFT method underestimates the interaction energy. Nevertheless, the magnitude of this energy is still higher than the chemisorption limit of 0.5 eV. Therefore, one can argue that the sensing mechanism of the Pb ions in pure aqueous solutions is governed by chemisorption processes with fast charge-transfer reactions near the hollow sites of the graphene. 

**Figure 8:**
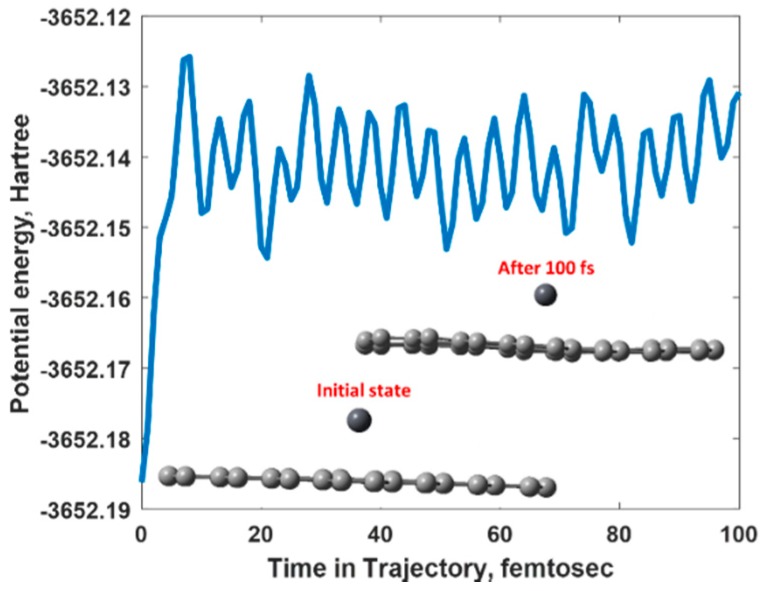
Potential energy trajectory for divalent Lead ion adsorbed onto graphene in the presence of aqueous solution.

To verify the stability of Cation-π bonding in the presence of water we performed ADMP simulations at room temperature. The potential energy trajectory represented in Figure 8 suggests that the adsorption of Pb^2+^ ion on graphene is very stable and the strong Cation-π interaction helps the Pb^2+^ ions to remain in the chemisorbed state on the graphene surface. In other words. Pb^2+^@graphene complex did not collapse with time. Such a dynamical stability will lead to reproducible resistance-time characteristics, which we observed in the experiment.

## 4. Conclusions

In summary, the behavior of Lead species at epitaxial graphene supported by Si-terminated 4H-SiC has been evaluated. By using two independent experimental methods, namely voltammetry and time-resolved electrical characterization we investigate the response of the epitaxial graphene under exposure to Pb-containing aqueous solutions. Raman mapped images provided by confocal Raman spectroscopy confirmed high quality of the epitaxial graphene and low density of point defects, indicating that adsorption events occur at the defect-free *sp*^2^-bonded carbon network. Our results indicated that Pb^2+^/Pb^0^ redox system at the graphene electrode surface is quasi-reversible. The results revealed a strong stripping response of Pb^2+^ with slope of linear regression of 2.3 × 10^3^ A·cm^−2^·mole^−1^·L and good linearity within concentration range between 10^−10^ M to 10^−6^ M. The adsorption behavior of Lead was further investigated with Raman spectroscopy. By monitoring of defect-related *D*-mode of graphene induced by Pb adsorption it was established that epitaxial graphene is an appropriate template providing high adsorption capacity towards Pb. The conductometric approach was found to be an effective tool for monitoring the presence of Lead ions in aqueous solutions, since graphene lateral resistor enables fast response and recovery. It was found that adsorption energy of Lead ions in the regime of low concentrations is higher than that at high concentrations, which is evidenced by the longer recovery time at the nanomolar concentrations. To elucidate the adsorption mechanisms, we also preformed DFT calculations. It was found that the electrochemical response of the epitaxial graphene to Lead is mainly regulated by a van der Waals interaction between neutral Lead atoms and graphene during a redox process, while the conductometric response is governed by the formation of stable charge-transfer complexes, which cause the increase in hole conductivity under Pb accumulation conditions. The current work provides deep insights into the fundamental understanding of the adsorption of Lead in different oxidative forms on epitaxial graphene and is an important step towards development of easy-to-use and portable sensing platform, which enables real-time determination of the toxic Lead in drinking water.

## Figures and Tables

**Table 1:** The parameters describing the electrochemical behavior of the Pb^2+^/Pb^0^ redox couple at room temperature at the epitaxial graphene.

Parameter	Anodic Process Pb^0^ − 2*e*→Pb^2+^	Cathodic Process Pb^2+^ + 2*e*→Pb^0^
Current density, mA/cm^2^	3.129	−0.852
Potential, V	−0.433	−0.669
Diffusion coefficient, 10^−3^ × cm^2^∙s^−1^	87.1	6.5
Electron transfer rate constant, 10^−2^ × cm∙s^−1^	0.57	1.46

**Table 2:** DFT-calculated parameters describing the solvent-mediated interaction between graphene and elemental Lead with and without consideration of the vdW correction.

Method	Interaction Energy, eV	Adsorption Energy, eV	Deformation Energy, eV	Distance, Å	Charge on Pb Atom		Dipole Moments, a.u.		
					Mulliken	Hirshfeld	*D*_x_	*D*_y_	*D*_z_
No vdW	−0.0049	−0.0069	0.0020	4.36	−0.00795	−0.01466	−0.00120	0.0000	0.055689
With vdW	0.3244	0.3163	0.0081	3.18	−0.04861	−0.14125	−0.00659	0.0000	0.382385

**Table 3:** DFT-calculated parameters describing the solvent-mediated interaction between graphene and divalent Lead ions.

Number of Pb Ions	Interaction Energy per Ion, eV	Adsorption Energy per Ion, eV	Deformation Energy, eV	Average Distance, Å	Charge on Pb^2+^ Ion		Dipole Moments, a.u.		
					Mulliken	Hirshfeld	*D*_x_	*D*_y_	*D*_z_
1	−1.6244	−1.6586	0.0342	2.45	1.3753	0.9826	−0.0021	−0.0012	1.375
	−0.5749 *	−0.6168 *	0.0418 *	2.44 *	1.5854 *	1.2984 *	−0.004 *	−0.001 *	1.284 *
2	−0.5759	−0.6254	0.0990	2.47	1.3732	0.9957	−0.0000	−0.0009	2.864
3	−0.3868	−0.4221	0.1059	2.51	1.4294	1.0497	−0.0006	−0.3469	4.228
4	−0.3221	−0.3496	0.1059	2.53	1.4723	1.1357	−0.0002	−0.0011	5.566

* vdW-corrected parameters.
